# Small ubiquitin-like modifier 4 (*SUMO4*) polymorphisms and Vogt-Koyanagi-Harada (VKH) syndrome in the Chinese Han population

**Published:** 2008-12-31

**Authors:** Shengping Hou, Peizeng Yang, Liping Du, Hongyan Zhou, Xiaomin Lin, Xiaoli Liu, Aize Kijlstra

**Affiliations:** 1 The First Affiliated Hospital, Chongqing Medical University, Chongqing Key Laboratory of Ophthalmology, Chongqing, P.R. China; 2 Zhongshan Ophthalmic Center, Sun Yat-sen University, Guangzhou, P.R. China; 3 Eye Research Institute Maastricht, Department of Ophthalmology, University Hospital Maastricht, Maastricht, the Netherlands

## Abstract

**Purpose:**

To examine whether small ubiquitin-like modifier 4 (*SUMO4*) polymorphisms were associated with Vogt-Koyanagi-Harada (VKH) syndrome in the Chinese Han population.

**Methods:**

Genotyping for *SUMO4* polymorphisms at G-847A, A-504G, A+163G, and C+438T loci was performed on 231 VKH patients and 302 controls using polymerase chain reaction restriction fragment length polymorphism.

**Results:**

A decreased frequency of *SUMO4* +438 TT genotype was found in VKH patients compared with healthy controls (p=0.009). However, the significance was lost after Bonferroni correction. Human leukocyte antigens (*HLA)-DR4* and *HLA-DRw53* were significantly associated with susceptibility to VKH syndrome (p=3.21×10^−16^ and 7.08×10^−5^, respectively). Stratification analysis based on *HLA-DR4* and *HLA-DRw53* did not show any associations between *SUMO4* polymorphisms and VKH syndrome, although there was a big difference in the percentage of certain allele and genotype frequencies between *HLA-DRw53* negative patients and controls. There was no significance in clinical findings and gender stratification analysis.

**Conclusions:**

*HLA-DR4* and *HLA-DRw53* are strongly associated with the susceptibility to VKH syndrome in the Chinese Han population. However, none of the currently known single nucleotide polymorphisms (SNPs) of *SUMO4* are associated with this syndrome.

## Introduction

Vogt-Koyanagi-Harada (VKH)syndrome is one of the most common uveitis entities in China [1]. It is characterized by a granulomatous panuveitis frequently in association with extraocular findings such as pleocytosis in the cerebrospinal fluid (CSF), dysacusis, alopecia, poliosis, and vitiligo [2-4]. Although the exact pathogenesis of VKH syndrome remains unclear, numerous studies have shown that immunogenetic factors are involved in the development of this syndrome. T cells autoreactive against tyrosinase family proteins are possibly involved in VKH syndrome. Meanwhile, genetic factors also play an important role in VKH syndrome as evidenced by the increased rates of this syndrome in pigmented groups [2], familial aggregation [5-7], and strong association with human leukocyte antigens (*HLA*)-*DR4* and *HLA-DRw53* in various ethnic groups including the Chinese and Japanese [8-10]. However, little is known about the genes that present susceptibility to the VKH syndrome except *HLA* [11-13].

Recently, studies have demonstrated that multiple autoimmune diseases may share common susceptibility genes by whole genome association and family based association studies [14-17]. Therefore, susceptibility genes associated with other autoimmune diseases may be candidates in the study of gene susceptibility to VKH syndrome, an autoimmune uveitis commonly seen in China. Small ubiquitin-like modifier 4 (*SUMO4*) is located on chromosome 6p25. Recently, certain *SUMO4* polymorphisms have been shown to be clearly associated with type 1 diabetes in multiple Asian populations [18,19] as well as with other autoimmune diseases [19-22], despite controversial observations in Caucasians [18,23-25]. *SUMO4* polymorphisms could also be involved in the pathogenesis of VKH syndrome, and this hypothesis was therefore the subject of the study presented here.

## Methods

### Subjects

Blood samples were collected from 231 Chinese Han VKH patients (128 males and 103 females) and 302 age- and sex-matched, unrelated Chinese Han healthy controls (164 males and 138 females), which were recruited from the Uveitis Study Center of the Zhongshan Ophthalmic Center, Sun Yat-sen University, Guangzhou, P.R. China and The First Affiliated Hospital, Chongqing Medical University, Chongqing, P.R. China. The institutional ethics committee of Zhongshan Ophthalmic Center, Sun Yat-sen University, Guangzhou, P.R. China approved this study, and informed consent was obtained from all tested subjects.

### DNA extraction

Genomic DNA samples were extracted and isolated from ethylene diamine tetraacetic acid (EDTA) anti-coagulated peripheral blood mononuclear cells (PBMCs) of VKH patients and healthy controls by a conventional salting out method. These DNA samples were diluted in PCR grade water and stored at −70 °C until used.

### Genotyping

Polymerase chain reaction (PCR) was performed using primers at G-847A locate (Forward, 5′-TCC CAA CCA ATA ATA GCA AGT CT-3′; Reverse, 5′-ATG CCT GGA TCA AAA CAC ACA-3′), A-504G locate (Forward, 5′- TGT GTG TTT TGA TCC AGG CAT TA-3′; Reverse, 5′-TGT TTT GCT CCT CTT TTC CTC TT-3′), A+163G locate (Forward, 5′-ATT GTG AAC CAC GGG GAT TGT TA-3′; Reverse, 5′-CAGCGTTCTGGAGTAAAGAAG-3′), and C+438T locate (Forward, 5′-ATA CCA GTT ACT TCA TGT ATA ATA GA-3′; Reverse, 5′-AGA TTA CTG CAT TCT CAA TTA G −3′). PCR products at G-847A (rs237026), A-504G (rs600739), A+163G (rs237025), and C+438T (rs237024) loci were incubated with SspI at 37 °C, Alw21I at 37 °C, MseI at 65 °C, and MnlI at 37 °C (MBI Fermentas, Vilnius, Lithuania) for at least 4 h, respectively. PCR fragments were separated on 3% agarose gels. Twenty percent of the PCR samples were directly sequenced to confirm the polymerase chain reaction restriction fragment length polymorphism (PCR-RFLP) results (Invitrogen Biotechnology Co., Guangzhou, China). *HLA-DR4* genotyping was performed using the PCR sequence specific primers (SSP) method as previously described [26]. *HLA-DRw53* genotyping was performed as previously described [27].

### Statistical analysis

The χ^2^ test was applied to analyze the Hardy–Weinberg equilibrium (HWE). The χ^2^ test or Fisher’s exact test was performed to compare the allelic, genotypic, and haplotypic distribution between VKH patients and healthy controls using version 12.0 of SPSS for Windows (SPSS Inc., Chicago, IL). Analysis of linkage disequilibrium (LD) of each SNP and haplotype was performed using the Haploview v3.32 program [28,29]. The p values were corrected using the Bonferroni correction to account for multiple testing. Sample sizes were estimated by Quanto 1.2 software (Department of Preventive Medicine, University of Southern California, Los Angles, CA).

## Results

### Descriptive data of the tested patients and controls

Detailed clinical findings of the enrolled VKH patients are shown in Table1. The average age of the VKH patients was 33.6±12.4 years and that of healthy controls was 35.4±12.0 years. No statistical difference was observed between VKH patients and controls in the distribution of age and gender (p>0.05).

**Table 1:** *HLA-DR4* and *HLA-DRw53 *distribution and clinical findings of patients with VKH syndrome.

**Characteristics**	**VKH Patients**			
	***HLA-DR4 +***	***HLA-DR4 -***	***HLA-DRw53 +***	***HLA-DRw53 -***
Number of patients (%)	179 (77.5%)	52 (22.5%)	203 (87.9%)	28 (12.1%)
Male	103 (80.5%)	25 (19.5%)	116 (90.6%)	12 (9.4%)
Female	76 (73.8%)	27 (26.2%)	87 (84.5%)	16 (15.5%)
Neck stiffness	74 (72.5%)	28 (27.5%)	89 (87.3%)	13 (12.7%)
Alopecia	26 (72.2%)	10 (27.8%)	30 (83.3%)	6 (16.7%)
Poliosis	65 (73.9%)	23 (26.1%)	78 (88.6%)	10 (11.4%)
Vitiligo	41 (74.5%)	14 (25.5%)	48 (87.3%)	7 (12.7%)
Dysacusia	57 (69.5%)	25 (30.5%)	70 (85.4%)	12 (14.6%)
Tinnitus	61 (84.7%)	11 (15.3%)	67 (93.1%)	5 (6.9%)
Scalp hypersensitivity	32 (74.4%)	11 (25.6%)	38 (88.4%)	5 (11.6%)

The age at onset (years±SD) for all VKH patients was 33.6±12.4 years.

### Single nucleotide polymorphism and haplotype analyses between polymorphisms of *SUMO4* and Vogt-Koyanagi-Harada syndrome

The distribution of genotype for each SNP including G-847A, A-504G, A+163G, and C+438T did not deviate from the HWE in VKH patients and healthy controls (p>0.05). A decreased frequency of *SUMO4* +438 TT genotype was observed in VKH patients compared with healthy controls (p=0.009, χ^2^=9.36). However, it did not remain significant after Bonferroni correction (Table 2).

**Table 2:** The comparison of allele and genotype frequencies for the four polymorphisms of *SUMO4 *gene in VKH patients and controls.

**SNPs**	**Genotype allele**	**VKH n=231 (%)**	**Controls n=302 (%)**	**χ^2^**	**p value**	**pc^a^ value**	**OR** **(95% CI)**
−847 G→A	GG	16 (7.1)	30 (10.1)	3.15	0.207	NS^b^	
	AG	104 (46.0)	116 (39.2)				
	AA	106 (46.9)	150 (50.7)				
	G	117 (30.1)	176 (29.7)	2.38	0.120	NS	1.0 (0.8–1.3)
	A	343 (69.9)	416 (70.3)				
−504 A→G	AA	54 (24.0)	66 (22.2)	0.25	0.885	NS	
	AG	107 (47.6)	146 (49.2)				
	GG	64 (28.4)	85 (28.6)				
	A	215 (47.8)	278 (46.8)	0.10	0.754	NS	1.0 (0.8–1.3)
	G	235 (52.2)	316 (53.2)				
+163 A→G	AA	107 (46.5)	145 (48.0)	1.05	0.591	NS	
	AG	107 (46.5)	130 (43.0)				
	GG	16 (7.0)	27 (8.9)				
	A	319 (69.7)	420 (69.5)	0.002	0.968	NS	1.0 (0.8–1.3)
	G	139 (30.3)	184 (30.5)				
+438 C→T	CC	107 (46.7)	135 (44.7)	9.36	0.009	NS	
	CT	107 (46.7)	122 (40.4)				
	TT	15 (6.6)	45 (14.9)				
	C	320 (70.2)	392 (64.9)	3.18	0.075	NS	1.3 (1.0–1.6)
	T	136 (29.8)	212 (35.1)				

^a^ Bonferroni corrected p value; ^b^Not significant.

Haplotype analysis using the Haploview 3.32 software showed that the four SNPs were in strong linkage (D’=84–91). A decreased frequency of *SUMO4* haplotype (−847A, −504G, +163A, and +438T) was observed in VKH patients compared with healthy controls (p=0.008, χ^2^=7.07). However, the significance was lost after Bonferroni correction (Table 3).

**Table 3:** The comparison of frequencies of *SUMO4* haplotypes in VKH Patients and controls.

**Haplotype**	**VKH n=231 (%)**	**Control n=302 (%)**	**χ^2^**	**p value**	**pc^a^ value**	**OR** **(95% CI)**
AGAC	228.2 (49.8)	274.6 (45.5)	1.99	0.158	NS^b^	1.2 (0.9–1.5)
GAGT	126.4 (27.6)	156.3 (25.9)	0.40	0.529	NS	1.1 (0.8–1.4)
AAAC	79.1 (17.3)	101.5 (16.8)	0.04	0.839	NS	1.0 (0.8–1.5)
AGAT	4.8 (1.0)	21.9 (3.6)	7.07	0.008	NS	0.2 (0.06–0.4)

^a^ Bonferroni corrected p value; ^b^Not significant.

### Stratification analysis of *SUMO4* polymorphisms with the status of *HLA-DR4*, *HLA-DRw53*, the clinical findings, and gender

Our study showed that the frequency of *HLA-DR4* was significantly increased in 231 VKH patients as compared with that in 302 healthy controls (77.5% versus 19.5%, p=3.21×10^−16^, χ^2^=66.67, OR=13.74, 95% CI=6.99–26.98). *HLA-DRw53* was also shown to be significantly associated with susceptibility to VKH syndrome in the Chinese Han population (87.9% versus 63.9%, p=7.08×10^−5^, χ^2^=15.79, OR=4.13, 95% CI=1.99–8.55). To test whether there was an influence of *HLA-DR4* and *DRw53* on the *SUMO4* association, stratification analysis was performed according to these parameters. The allele and genotype frequencies of the four SNPs of *SUMO4* were not different between *HLA-DR4* positive patients and *HLA-DR4* positive controls and between *HLA-DR4* negative patients and *HLA-DR4* negative controls (Table 4). Similar results were also observed in *HLA-DRw53* stratification analysis (Table 5). However, a big difference was observed in *HLA-DRw53* negative patients compared with *HLA-DRw53* negative controls (G-847A: AA genotype, *HLA-DRw53*- patients versus *HLA-DRw53*- controls: 40.7% versus 59.4%, AG genotype, *HLA-DRw53*- patients versus *HLA-DRw53*- controls: 48.1% versus 31.1%; A-504G: AA genotype, *HLA-DRw53*- patients versus *HLA-DRw53*- controls: 40.8% versus 21.5%, A allele, *HLA-DRw53*- patients versus *HLA-DRw53*- controls: 59.3% versus 43.9%; A+163G: AA genotype, *HLA-DRw53*- patients versus *HLA-DRw53*- controls: 39.3% versus 56.9%, AG genotype, *HLA-DRw53*- patients versus *HLA-DRw53*- controls: 50.0% versus 33.0%; Table 5).

**Table 4:** Stratification analysis for *HLA-DR4* and *SUMO4* polymorphisms and the comparison of frequencies of *SUMO4 *allele and genotype in* HLA-DR4*+ patients versus *HLA-DR4*+ controls and *HLA-DR4*- patients versus *HLA-DR4*- controls.

**SNPs**	**Genotype allele**	***HLA-DR4*+ Patients n=179 (%)**	***HLA-DR4*+ Controls n=59 (%)**	**p value**	**pc^a^ value**	***HLA-DR4*- Patients n=52 (%)**	***HLA-DR4*- Controls n=243 (%)**	**p value**	**pc^a^ value**
−847 G→A	GG	12 (6.8)	10 (17.2)	0.041	NS^b^	4 (8.0)	20(8.4)	0.757	NS
	AG	81 (46.0)	20 (34.5)			23 (46.0)	96 (40.3)		
	AA	83 (47.2)	28 (48.3)			23 (46.0)	122 (51.3)		
	G	105 (29.8)	40 (34.5)	0.347	NS	31 (31.0)	136 (28.6)	0.627	NS
	A	247 (70.2)	76 (65.5)			69 (69.0)	340 (71.4)		
−504 A→G	AA	40 (22.9)	11 (19.0)	0.656	NS	14 (28.0)	55 (23.0)	0.696	NS
	AG	85 (48.6)	27 (46.6)			22 (44.0)	119 (49.8)		
	GG	50 (28.6)	20 (34.5)			14 (28.0)	65 (27.2)		
	A	165 (47.1)	49 (42.2)	0.359	NS	50 (50.0)	249 (52.1)	0.703	NS
	G	185 (52.9)	67 (57.8)			50 (50.0)	229 (47.9)		
+163 A→G	AA	84 (46.9)	29 (49.2)	0.820	NS	23 (45.1)	116 (47.7)	0.872	NS
	AG	83 (46.4)	25 (42.4)			24 (47.1)	105 (43.2)		
	GG	12 (6.7)	5 (8.5)			4 (7.8)	22 (9.1)		
	A	251 (70.1)	83 (70.3)	0.963	NS	70 (68.6)	337 (69.3)	0.887	NS
	G	107 (29.9)	35 (29.7)			32 (31.4)	149 (30.7)		
+438 C→T	CC	82 (46.3)	29 (49.2)	0.089	NS	25 (48.1)	106 (43.6)	0.562	NS
	CT	85 (48.0)	22 (37.3)			22 (42.3)	100 (41.2)		
	TT	10 (5.6)	8 (13.6)			5 (9.6)	37 (15.2)		
	C	249 (70.3)	80 (67.8)	0.603	NS	72 (69.2)	312 (64.2)	0.328	NS
	T	105 (29.7)	38 (32.2)			32 (30.8)	174 (35.8)		

^a^ Bonferroni corrected p value; ^b^Not significant.

**Table 5:** Stratification analysis for *HLA-DRw53* and *SUMO4* polymorphisms and the comparison of frequencies of *SUMO4 *allele and genotype in* HLA-DRw53*+ patients versus *HLA-DRw53*+ controls and *HLA-DRw53*- patients versus *HLA-DRw53*- controls.

**SNPs**	**Genotype allele**	***HLA-DRw53*+ Patients n=203 (%)**	***HLA-DRw53*+ Controls n=193 (%)**	**p value**	**pc^a^ value**	***HLA-DRw53*- Patients n=28 (%)**	***HLA-DRw53*- Controls n=109 (%)**	**p value**	**pc^a^ value**
−847 G→A	GG	13 (6.5)	20 (10.5)	0.368	NS^b^	3 (11.1)	10 (9.4)	0.200	NS
	AG	91 (45.7)	83 (43.7)			13 (48.1)	33 (31.1)		
	AA	95 (47.7)	87 (45.8)			11 (40.7)	63 (59.4)		
	G	117 (29.4)	123 (32.4)	0.370	NS	19 (35.2)	53 (25.0)	0.133	NS
	A	281 (70.6)	257 (67.6)			35 (64.8)	159 (75.0)		
−504 A→G	AA	43 (21.7)	43 (22.6)	0.742	NS	11 (40.8)	23 (21.5)	0.114	NS
	AG	97 (49.0)	98 (51.6)			10 (37.0)	48 (44.9)		
	GG	58 (29.3)	49 (25.8)			6 (22.2)	36 (33.6)		
	A	183 (46.2)	184 (48.4)	0.538	NS	32 (59.3)	94 (43.9)	0.044	NS
	G	213 (53.8)	196 (51.6)			22 (40.7)	120 (56.1)		
+163 A→G	AA	96 (47.5)	83 (43.0)	0.590	NS	11 (39.3)	62 (56.9)	0.216	NS
	AG	93 (46.0)	94 (48.7)			14 (50.0)	36 (33.0)		
	GG	13 (6.4)	16 (8.3)			3 (10.7)	11 (10.1)		
	A	285 (70.5)	260 (67.4)	0.333	NS	36 (64.3)	160 (73.4)	0.178	NS
	G	119 (29.5)	126 (32.6)			20 (35.7)	58 (26.6)		
+438 C→T	CC	95 (47.3)	82 (42.5)	0.035	NS	12 (42.9)	53 (48.6)	0.613	NS
	CT	95 (47.3)	86 (44.6)			12 (42.9)	36 (33.0)		
	TT	11 (5.5)	25 (13.0)			4 (14.3)	20 (18.3)		
	C	285 (70.9)	250 (64.8)	0.065	NS	36 (64.3)	142 (65.1)	0.905	NS
	T	117 (29.1)	136 (35.2)			20 (35.7)	76 (34.9)		

^a^Bonferroni corrected p value; ^b^Not significant.

Stratification analysis was also performed according to clinical findings including neck stiffness, tinnitus, alopecia, poliosis, dysacusis, scalp hypersensitivity, and vitiligo. No association was found between the four SNPs and any extraocular findings. The analysis of gender stratification also showed no association of *SUMO4* polymorphisms with VKH syndrome.

## Discussion

In this study, we examined the association of *SUMO4* polymorphisms with VKH syndrome in the Chinese Han population. Our results failed to find an association between *SUMO4* polymorphisms and VKH syndrome even after stratification for *HLA-DR4*, *HLA-DRw53*, clinical features, and gender.

*SUMO4* has been shown to be involved in the regulation of *NF-кB*, an important transcription factor in autoimmune diseases. It has been reported that the *SUMO4* A+163G (M55V) polymorphism is an essential polymorphism involved in regulating its own sumoylation, and it has been shown to be associated with certain autoimmune diseases such as type 1 diabetes, autoimmune thyroid disease, and rheumatoid arthritis without amyloidosis [19]. These results suggest that this polymorphism could be a susceptibility gene shared by certain autoimmune diseases, although conflicting data have been reported in Sjögren’s syndrome [19]. The identification of a general susceptibility gene for several autoimmune diseases could make an important contribution to the understanding of the pathogenesis and modulation of these diseases. The question was therefore raised whether the *SUMO4* A+163G polymorphism was also associated with VKH syndrome. This study was designed to clarify this issue. We strictly chose the patients who were definitely diagnosed with VKH syndrome according to the revised criteria [30] to exclude the influence of misdiagnosis. As ethnic confounding could also influence the association results, only VKH patients with Chinese Han nationality as well as age- and sex-matched controls with the same nationality were enrolled in this study. The frequency of the +163G allele in the control population presented in our study is similar with that in the Chinese population reported by Li et al. [31] and in the Japanese population reported by Noso et al. [20]. Meanwhile, a power analysis of the study population showed that our sample size was large enough to detect a possible association. Unexpectedly, we failed to find an association of the *SUMO4* A+163G polymorphism with VKH syndrome. This suggests that this polymorphism may not be involved in the development of susceptibility to VKH syndrome.

Others SNPs including G-847A, A-504G, and C+438T polymorphisms have been identified by direct sequencing of the whole *SUMO4* gene in the Japanese population [20,32]. Our previous results showed an association of *SUMO4* C+438T polymorphism with Behcet’s disease [22], another common uveitis entity observed in China. The present study also failed to show any association of the *SUMO4* G-847A, A-504G, and C+438T polymorphisms with VKH syndrome. This difference may result from the different features of these two uveitis entities. One of the striking features of Behcet’s disease is its characteristic non-granulomatous inflammation while VKH syndrome is in fact a granulomatous inflammation [33].

Like *HLA-DR4, HLA-DRw53* have been demonstrated to be strongly associated with VKH syndrome. Therefore, genotyping of *HLA-DR4* and *HLA-DRw53* was performed. The association of *HLA-DR4* and *HLA-DRw53* with VKH syndrome was extremely strong in this study. The results were generally consistent with those previously reported in Chinese [4,8,34] and Spanish patients [35]. Furthermore, stratification analysis according to *HLA-DR4* and *HLA-DRw53* did not show any association of *SUMO4* with VKH syndrome in our study. This result is consistent with the previous studies that *SUMO4* M55V polymorphism was independent of the *HLA* class II haplotype [19,32], which is located on the same chromosome 6 as *SUMO4*.

It is worthy to point out that there was a big difference in the percentages of certain alleles and genotypes between *HLA-DRw53* negative patients and controls, although the difference did not reach statistical significance. As the sample size of *HLA-DRw53* negative is small (28 patients), it is necessary to further test the association of *SUMO4* polymorphisms with *HLA-DRw53* negative patients using larger samples.

In conclusion, we failed to detect an association of *SUMO4* polymorphisms with VKH syndrome in Chinese Han population. In agreement with earlier studies, we found a strong association of *HLA-DR4* and *HLA-DRw53* with susceptibility to VKH syndrome. A big but insignificant difference of allele and genotype frequency was noted in *HLA-DRw53* negative patients when compared with *HLA-DRw53* negative controls. Further studies are necessary to elucidate the association of *SUMO4* polymorphisms with VKH syndrome in a *HLA-DRw53* negative population using larger samples.

## References

[1] Yang P, Zhang Z, Zhou H, Li B, Huang X, Gao Y, Zhu L, Ren Y, Klooster J, Kijlstra A (2005). Clinical patterns and characteristics of uveitis in a tertiary center for uveitis in China.. Curr Eye Res.

[2] Moorthy RS, Inomata H, Rao NA (1995). Vogt-Koyanagi-Harada syndrome.. Surv Ophthalmol.

[3] Forster DJ, Cano MR, Green RL, Rao NA (1990). Echographic features of the Vogt-Koyanagi-Harada syndrome.. Arch Ophthalmol.

[4] Yang P, Ren Y, Li B, Fang W, Meng Q, Kijlstra A (2007). Clinical characteristics of Vogt-Koyanagi-Harada syndrome in Chinese patients.. Ophthalmology.

[5] Ishikawa A, Shiono T, Uchida S (1994). Vogt-Koyanagi-Harada disease in identical twins.. Retina.

[6] Rutzen AR, Ortega-Larrocea G, Schwab IR, Rao NA (1995). Simultaneous onset of Vogt-Koyanagi-Harada syndrome in monozygotic twins.. Am J Ophthalmol.

[7] Itho S, Kurimoto S, Kouno T (1992). Vogt-Koyanagi-Harada disease in monozygotic twins.. Int Ophthalmol.

[8] Zhao M, Jiang Y, Abrahams IW (1991). Association of HLA antigens with Vogt-Koyanagi-Harada syndrome in a Han Chinese population.. Arch Ophthalmol.

[9] Zhang XY, Wang XM, Hu TS (1992). Profiling human leukocyte antigens in Vogt-Koyanagi-Harada syndrome.. Am J Ophthalmol.

[10] Islam SM, Numaga J, Fujino Y, Hirata R, Matsuki K, Maeda H, Masuda K (1994). HLA class II genes in Vogt-Koyanagi-Harada disease.. Invest Ophthalmol Vis Sci.

[11] Du L, Yang P, Hou S, Lin X, Zhou H, Huang X, Wang L, Kijlstra A (2008). Association of the CTLA-4 gene with Vogt-Koyanagi-Harada syndrome.. Clin Immunol.

[12] Horie Y, Takemoto Y, Miyazaki A, Namba K, Kase S, Yoshida K, Ota M, Hasumi Y, Inoko H, Mizuki N, Ohno S (2006). Tyrosinase gene family and Vogt-Koyanagi-Harada disease in Japanese patients.. Mol Vis.

[13] Horie Y, Kitaichi N, Takemoto Y, Namba K, Yoshida K, Hirose S, Hasumi Y, Ota M, Inoko H, Mizuki N, Ohno S (2007). Polymorphism of IFN-gamma gene and Vogt-Koyanagi-Harada disease.. Mol Vis.

[14] Wellcome Trust Case Control Consortium (2007). Genome-wide association study of 14,000 cases of seven common diseases and 3,000 shared controls.. Nature.

[15] Cargill M, Schrodi SJ, Chang M, Garcia VE, Brandon R, Callis KP, Matsunami N, Ardlie KG, Civello D, Catanese JJ, Leong DU, Panko JM, McAllister LB, Hansen CB, Papenfuss J, Prescott SM, White TJ, Leppert MF, Krueger GG, Begovich AB (2007). A large-scale genetic association study confirms IL12B and leads to the identification of IL23R as psoriasis-risk genes.. Am J Hum Genet.

[16] Burton PR, Clayton DG, Cardon LR, Craddock N, Deloukas P, Duncanson A, Kwiatkowski DP, McCarthy MI, Ouwehand WH, Samani NJ, Todd JA, Donnelly P, Barrett JC, Davison D, Easton D, Evans DM, Leung HT, Marchini JL, Morris AP, Spencer CC, Tobin MD, Attwood AP, Boorman JP, Cant B, Everson U, Hussey JM, Jolley JD, Knight AS, Koch K, Meech E, Nutland S, Prowse CV, Stevens HE, Taylor NC, Walters GR, Walker NM, Watkins NA, Winzer T, Jones RW, McArdle WL, Ring SM, Strachan DP, Pembrey M, Breen G, St Clair D, Caesar S, Gordon-Smith K, Jones L, Fraser C, Green EK, Grozeva D, Hamshere ML, Holmans PA, Jones IR, Kirov G, Moskivina V, Nikolov I, O'Donovan MC, Owen MJ, Collier DA, Elkin A, Farmer A, Williamson R, McGuffin P, Young AH, Ferrier IN, Ball SG, Balmforth AJ, Barrett JH, Bishop TD, Iles MM, Maqbool A, Yuldasheva N, Hall AS, Braund PS, Dixon RJ, Mangino M, Stevens S, Thompson JR, Bredin F, Tremelling M, Parkes M, Drummond H, Lees CW, Nimmo ER, Satsangi J, Fisher SA, Forbes A, Lewis CM, Onnie CM, Prescott NJ, Sanderson J, Matthew CG, Barbour J, Mohiuddin MK, Todhunter CE, Mansfield JC, Ahmad T, Cummings FR, Jewell DP, Webster J, Brown MJ, Lathrop MG, Connell J, Dominiczak A, Marcano CA, Burke B, Dobson R, Gungadoo J, Lee KL, Munroe PB, Newhouse SJ, Onipinla A, Wallace C, Xue M, Caulfield M, Farrall M, Barton A, Bruce IN, Donovan H, Eyre S, Gilbert PD, Hilder SL, Hinks AM, John SL, Potter C, Silman AJ, Symmons DP, Thomson W, Worthington J, Dunger DB, Widmer B, Frayling TM, Freathy RM, Lango H, Perry JR, Shields BM, Weedon MN, Hattersley AT, Hitman GA, Walker M, Elliott KS, Groves CJ, Lindgren CM, Rayner NW, Timpson NJ, Zeggini E, Newport M, Sirugo G, Lyons E, Vannberg F, Hill AV, Bradbury LA, Farrar C, Pointon JJ, Wordsworth P, Brown MA, Franklyn JA, Heward JM, Simmonds MJ, Gough SC, Seal S, Stratton MR, Rahman N, Ban M, Goris A, Sawcer SJ, Compston A, Conway D, Jallow M, Newport M, Sirugo G, Rockett KA, Bumpstead SJ, Chaney A, Downes K, Ghori MJ, Gwilliam R, Hunt SE, Inouye M, Keniry A, King E, McGinnis R, Potter S, Ravindrarajah R, Whittaker P, Widden C, Withers D, Cardin NJ, Davison D, Ferreira T, Pereira-Gale J, Hallgrimsdo'ttir IB, Howie BN, Su Z, Teo YY, Vukcevic D, Bentley D, Brown MA, Compston A, Farrall M, Hall AS, Hattersley AT, Hill AV, Parkes M, Pembrey M, Stratton MR, Mitchell SL, Newby PR, Brand OJ, Carr-Smith J, Pearce SH, McGinnis R, Keniry A, Deloukas P, Reveille JD, Zhou X, Sims AM, Dowling A, Taylor J, Doan T, Davis JC, Savage L, Ward MM, Learch TL, Weisman MH, Brown M (2007). Association scan of 14,500 nonsynonymous SNPs in four diseases identifies autoimmunity variants.. Nat Genet.

[17] Duerr RH, Taylor KD, Brant SR, Rioux JD, Silverberg MS, Daly MJ, Steinhart AH, Abraham C, Regueiro M, Griffiths A, Dassopoulos T, Bitton A, Yang H, Targan S, Datta LW, Kistner EO, Schumm LP, Lee AT, Gregersen PK, Barmada MM, Rotter JI, Nicolae DL, Cho JH (2006). A genome-wide association study identifies IL23R as an inflammatory bowel disease gene.. Science.

[18] Guo D, Li M, Zhang Y, Yang P, Eckenrode S, Hopkins D, Zheng W, Purohit S, Podolsky RH, Muir A, Wang J, Dong Z, Brusko T, Atkinson M, Pozzilli P, Zeidler A, Raffel LJ, Jacob CO, Park Y, Serrano-Rios M, Larrad MT, Zhang Z, Garchon HJ, Bach JF, Rotter JI, She JX, Wang CY (2004). A functional variant of SUMO4, a new I kappa B alpha modifier, is associated with type 1 diabetes.. Nat Genet.

[19] Tsurumaru M, Kawasaki E, Ida H, Migita K, Moriuchi A, Fukushima K, Fukushima T, Abiru N, Yamasaki H, Noso S, Ikegami H, Awata T, Sasaki H, Eguchi K (2006). Evidence for the role of small ubiquitin-like modifier 4 as a general autoimmunity locus in the Japanese population.. J Clin Endocrinol Metab.

[20] Noso S, Fujisawa T, Kawabata Y, Asano K, Hiromine Y, Fukai A, Ogihara T, Ikegami H (2007). Association of small ubiquitin-like modifier 4 (SUMO4) variant, located in IDDM5 locus, with type 2 diabetes in the Japanese population.. J Clin Endocrinol Metab.

[21] Lin HY, Wang CL, Hsiao PJ, Lu YC, Chen SY, Lin KD, Hsin SC, Hsieh MC, Shin SJ (2007). SUMO4 M55V variant is associated with diabetic nephropathy in type 2 diabetes.. Diabetes.

[22] Hou S, Yang P, Du L, Zhou H, Lin X, Liu X, Kijlstra A (2008). SUMO4 gene polymorphisms in Chinese Han patients with Behcet's disease.. Clin Immunol.

[23] Owerbach D, Pina L, Gabbay KH (2004). A 212-kb region on chromosome 6q25 containing the TAB2 gene is associated with susceptibility to type 1 diabetes.. Diabetes.

[24] Qu H, Bharaj B, Liu XQ, Curtis JA, Newhook LA, Paterson AD, Hudson TJ, Polychronakos C (2005). Assessing the validity of the association between the SUMO4 M55V variant and risk of type 1 diabetes.. Nat Genet.

[25] Smyth DJ, Howson JM, Lowe CE, Walker NM, Lam AC, Nutland S, Hutchings J, Tuomilehto-Wolf E, Tuomilehto J, Guja C, Ionescu-Tirgoviste C, Undlien DE, Ronningen KS, Savage D, Dunger DB, Twells RC, McArdle WL, Strachan DP, Todd JA (2005). Assessing the validity of the association between the SUMO4 M55V variant and risk of type 1 diabetes.. Nat Genet.

[26] Zetterquist H, Olerup O (1992). Identification of the HLA-DRB1*04, -DRB1*07, and -DRB1*09 alleles by PCR amplification with sequence-specific primers (PCR-SSP) in 2 hours.. Hum Immunol.

[27] Richeldi L, Sorrentino R, Saltini C (1993). HLA-DPB1 glutamate 69: a genetic marker of beryllium disease.. Science.

[28] Barrett JC, Fry B, Maller J, Daly MJ (2005). Haploview: analysis and visualization of LD and haplotype maps.. Bioinformatics.

[29] Qin ZS, Niu T, Liu JS (2002). Partition-ligation-expectation-maximization algorithm for haplotype inference with single-nucleotide polymorphisms.. Am J Hum Genet.

[30] Read RW, Holland GN, Rao NA, Tabbara KF, Ohno S, Arellanes-Garcia L, Pivetti-Pezzi P, Tessler HH, Usui M (2001). Revised diagnostic criteria for Vogt-Koyanagi-Harada disease: report of an international committee on nomenclature.. Am J Ophthalmol.

[31] Li H, Gao L, Shen Z, Li CY, Li K, Li M, Lv YJ, Li CX, Gao TW, Liu YF (2008). Association study of NFKB1 and SUMO4 polymorphisms in Chinese patients with psoriasis vulgaris.. Arch Dermatol Res.

[32] Noso S, Ikegami H, Fujisawa T, Kawabata Y, Asano K, Hiromine Y, Tsurumaru M, Sugihara S, Lee I, Kawasaki E, Awata T, Ogihara T (2005). Genetic heterogeneity in association of the SUMO4 M55V variant with susceptibility to type 1 diabetes.. Diabetes.

[33] Inomata H, Minei M, Taniguchi Y, Nishimura F (1983). Choroidal neovascularization in long-standing case of Vogt-Koyanagi-Harada disease.. Jpn J Ophthalmol.

[34] Xiao T, Jiang Y, You X (1997). The association of HLA-DR4 gene subtypes with Vogt-Koyanagi-Harada syndrome.. Zhonghua Yan Ke Za Zhi.

[35] Weisz JM, Holland GN, Roer LN, Park MS, Yuge AJ, Moorthy RS, Forster DJ, Rao NA, Terasaki PI (1995). Association between Vogt-Koyanagi-Harada syndrome and HLA-DR1 and -DR4 in Hispanic patients living in southern California.. Ophthalmology.

